# Template-synthesis of a poly(ionic liquid)-derived Fe_1−*x*_S/nitrogen-doped porous carbon membrane and its electrode application in lithium–sulfur batteries†

**DOI:** 10.1039/d1ma00441g

**Published:** 2021-06-25

**Authors:** Sadaf Saeedi Garakani, Dongjiu Xie, Atefeh Khorsand Kheirabad, Yan Lu, Jiayin Yuan

**Affiliations:** Department of Materials and Environmental Chemistry, Stockholm University Stockholm 10691 Sweden jiayin.yuan@mmk.su.se; Department for Electrochemical Energy Storage, Helmholtz-Zentrum Berlin für Materialien und Energie Hahn-Meitner Platz 1 Berlin 14109 Germany yan.lu@helmholtz-berlin.de; Institute of Chemistry, University of Potsdam 14476 Potsdam Germany

## Abstract

This study deals with the facile synthesis of Fe_1−*x*_S nanoparticle-containing nitrogen-doped porous carbon membranes (denoted as Fe_1−*x*_S/N-PCMs) *via* vacuum carbonization of hybrid porous poly(ionic liquid) (PIL) membranes, and their successful use as a sulfur host material to mitigate the shuttle effect in lithium–sulfur (Li–S) batteries. The hybrid porous PIL membranes as the sacrificial template were prepared *via* ionic crosslinking of a cationic PIL with base-neutralized 1,1′-ferrocenedicarboxylic acid, so that the iron source was molecularly incorporated into the template. The carbonization process was investigated in detail at different temperatures, and the chemical and porous structures of the carbon products were comprehensively analyzed. The Fe_1−*x*_S/N-PCMs prepared at 900 °C have a multimodal pore size distribution with a satisfactorily high surface area and well-dispersed iron sulfide nanoparticles to physically and chemically confine the LiPSs. The sulfur/Fe_1−*x*_S/N-PCM composites were then tested as electrodes in Li–S batteries, showing much improved capacity, rate performance and cycle stability, in comparison to iron sulfide-free, nitrogen-doped porous carbon membranes.

## Abstract 2

We designed a novel Fe_1−*x*_S/N-doped porous carbon membrane and applied it as a sulfur host material to moderate the shuttle effect in lithium–sulfur batteries.

## Graphical abstract

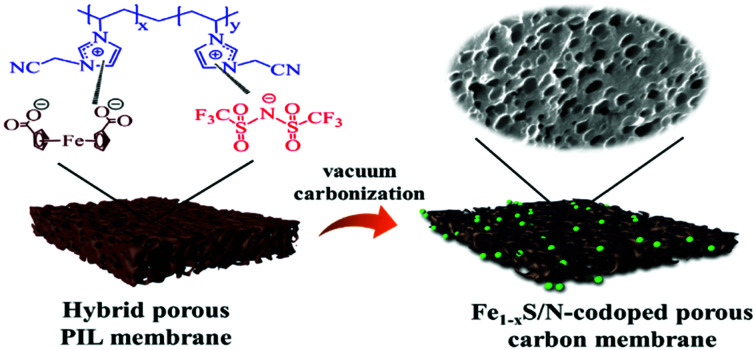

## Introduction

Over the past decade, heteroatom-doped porous carbons have attracted a great deal of attention due to their unique and tuneable physical and chemical properties from the atomic to macroscopic scale. For instance, the specific surface area, hierarchical pore structure, electron density, electric conductivity, and oxidation resistance can be mentioned, demonstrating a vast range of physicochemical attributes accessible to satisfy various purposes.^1–3^ The multimodal and hierarchical pore sizes of heteroatom-doped porous carbons relating to the pore size distribution from macropores (>50 nm) down to mesopores (2–50 nm) and micropores (<2 nm) can be developed to well balance the diffusion resistance of reactants toward and away from the active sites, and a high surface area to accommodate active sites.

To be highlighted is the doping of carbons with nitrogen (N), which is to date the most popularly used heteroatom to dope carbon due to its wide presence in various organic compounds to be used as carbon precursors, and its similar atomic size to a carbon atom which facilitates the easy formation of C–N covalent bonds inside the carbon matrix. The doped N atoms have the capability of altering the physicochemical characteristics of carbon materials and supplying carbon materials with target-specific functions, such as high conductivity, oxidation resistance, and catalytic activities.^4^ The application scope of nitrogen-doped porous carbons (NPCs) has been expanded drastically in the past two decades. They have been utilized in batteries,^5,6^ fuel cells,^7^ catalysis,^8^ membrane separation,^9^ and supercapacitors.^10^ Particularly, NPCs have received considerable interest in lithium-ion batteries and lithium–sulfur (Li–S) batteries.^11,12^ Tao *et al.* showed that N-doped carbons enhanced the Li_2_S conversion in Li–S batteries. Moreover, the existence of pyridinic N atoms in porous carbon was reported to be highly effective in the capture of lithium polysulfides and the further reduction towards Li_2_S.^13^ Wei *et al.* demonstrated that the hierarchically structured NPCs reduced the Li^+^ diffusion pathway and increased the active sites for Li^+^ storage in lithium-ion batteries. Furthermore, such NPCs could improve the electrical conductivity and modify volume alterations that occur in the cycling tests.^14^ To be highlighted here is the nitrogen-doped porous carbon membrane (N-PCM), which is a unique form of NPCs containing a hierarchical interconnected porous structure in a membrane state. There has been recently increasing interest in synthesizing and applying N-PCMs as an electrode in various electrochemical devices.^15^

The chemical structure of the precursor for the synthesis of NPCs plays a crucial role in determining the final shape, carbonization yield, chemical composition, and physicochemical properties. Dai and Antonietti *et al.* are the pioneers who synthesized NPCs based on nitrile-functionalized ionic liquids (ILs).^16,17^ Thereafter, ILs have been receiving considerable interest for their use in the synthesis of NPCs with a high nitrogen content, elevated pyrolytic yield, and high specific surface area.^18^ Our group applied poly(ionic liquid) (PIL), the polymerization product of ILs, as a precursor to produce porous carbons in a variety of defined shapes.^19^ In PILs, the polymer nature allows them to be processed into well-defined structures or shapes, and the cation–anion pair is one of the critical factors in creating the micro-/mesopores.^19^ Compared to other classes of polymers, PILs have some unique advantages. First and foremost, PILs can be of high thermal stability up to 450 °C and thus their carbonization yield is usually higher than their neutral counterparts.^20^ Secondly, using rich heteroatoms in the PIL's chemical structure helps control the heteroatom type and content, mainly nitrogen but also S, B and P, in the final carbon product. Thirdly, PIL structures in various shapes, such as membranes, nanotubes, and spheres, due to a high carbonization yield, can be better preserved during the carbonization at high temperature, *e.g.* at 1000 °C.^15^ Thus, PILs are a good platform to produce shaped porous carbon structures.

One of the important usages of NPCs is their energy application. Li–S batteries are a type of rechargeable batteries with noticeable features of high specific energy density, low materials cost, environmentally friendliness, and abundant availability of sulfur, making them promising as a future energy storage set.^21^ However, some obstacles, including limited electrical conductivity of sulfur, the rapid capacity declining induced by the dissolution of lithium polysulfides (LiPSs), and significant volume alterations through discharging/charging cycles hinder their practical usage.^22–24^ To mitigate these problems, NPCs are one of the promising solutions due to their high electronic conductivity and the hierarchically porous structure to buffer volume alterations.^25^ To this end, some metal compounds, in particular transition metal sulfides, nitrides, and carbides, reveal a strong chemical bonding potential with LiPSs, which can improve the diffusion and catalytic reduction of soluble LiPSs.^26,27^ Recently, metal sulfides M_*x*_S, such as FeS,^5^ FeS_2_,^28,29^ NiS,^30^ and WS_2_^31^ were investigated as cathodes for Li–S batteries. The results disclosed that high LiPSs adsorption and following redox reaction on these catalysts enhanced the sulfur usage. Their structural defects and poor electrical conductivity can be solved to a large extent by incorporation into NPCs that are conductive when produced at high temperatures,^21,32^ making the M_*x*_S/NPC system attractive cathode materials for Li–S batteries.

In this contribution, we succeeded in synthesizing **N-PCMs** containing iron sulfide nanoparticles (termed **Fe1−xS**/**N-PCMs**), *via* vacuum carbonization of an iron-containing porous hybrid PIL membrane as a sacrificial template at 900 °C. The **Fe1−xS**/**N-PCM** sample carrying a specific surface area of 274 m^2^ g^−1^, 5.0 wt% of N and 15 wt% of well-dispersed iron sulfide nanoparticles of 25 ± 7 nm in size was successfully applied as the cathode in Li–S batteries. In comparison with iron sulfide-free **N-PCMs**, the **Fe1−xS**/**N-PCMs** were found to be effective in decreasing the shuttling behaviour of LiPSs, showing a higher capacity and extended cycle life.

## Materials and methods

### Materials

1-Vinylimidazole (99%) and sodium tetrafluoroborate were obtained from Alfa Aesar. Potassium hexafluorophosphate was purchased from Acros Organics. 1,1′-Ferrocene dicarboxylic acid was bought from Energy Chemical. Bromoacetonitrile (95%) was purchased from TCI Europe. Lithium bis(trifluoromethane sulfonyl)imide (LiTFSI, 99.95%) was purchased from Io-li-tec. Lithium nitrate (LiNO_3_), polyvinylidene fluoride (PVDF), *N*-methyl-2-pyrrolidone (NMP), 1,2-dimethoxyethane (DME), and 1,3-dioxolane (DOL) were purchased from Sigma-Aldrich. Sulfur powder was purchased from Alfa Aesar. All chemicals were used without any further purification. Solvents were of analytical grade.

### Synthesis of the PIL

The polymer precursor with Br^−^ anions, poly(1-cyanomethyl-3-vinylimidazoulim bromide) (PCMVImBr) was synthesized in reference to our earlier published procedure.^19^ Its chemical structure was characterized utilizing proton nuclear magnetic resonance (^1^H-NMR), as demonstrated in Fig. S1 (ESI†). Poly(1-cyanomethyl-3-vinylimidazoluim bis(trifluoromethane sulfonyl)imide) (PCMVImTFSI) was made by anion exchange reaction between PCMVImBr and LiTFSI in their aqueous solution. In a standard anion-exchange procedure, an aqueous solution of LiTFSI was added dropwise to an aqueous solution of PCMVImBr at a concentration of 1 wt%. The final TFSI/Br molar ratio was set as 1.15/1. The precipitate was washed with pure water several times and dried at 70 °C under vacuum until reaching a constant weight.

### Fabrication of hybrid porous PIL membranes

0.200 g of the as-synthesized PCMVImTFSI and 0.068 g of 1,1′-ferrocenedicarboxylic acid (**FDA**) (the imidazolium/carboxylate molar ratio is 1/1) were thoroughly dissolved in 2 ml of DMSO till a homogenous solution was gained. The solution was then poured onto a pre-cleaned glass plate and dried at 80 °C for 2 h. The obtained film sticking to the glass substrate was immersed into a 0.25 wt% aqueous ammonia solution for 2 h to generate a porous polymeric membrane. The polymeric membrane was then peeled off from its underneath glass substrate and washed with pure water several times. It was finally dried under ambient conditions until reaching a constant weight and stored at room temperature for further utilization.

### Carbonization process

In a typical experiment, the carbonization was performed by the following procedure: the polymeric membrane was heated to 300 °C at a heating rate of 5 °C min^−1^ and was maintained at this temperature for 1 h; then, it was heated to 600 °C at a rate of 5 °C min^−1^ and was kept again for 1 h; finally, it was heated to 900 °C at a rate of 1 °C min^−1^ and was retained for 1 h. Afterwards, the furnace was cooled down to ambient temperature in 6 h. During the whole process, the furnace vacuum was kept constant at 13 mbar. A similar procedure was followed for the preparation of other NPCs with various carbonization temperatures.

### Preparation of the sulfur composite cathode

Firstly, sublimed sulfur powder was ground with the **Fe1−xS**/**N-PCMs-900** membrane at a mass ratio of 7 : 3 in a mortar. After grinding for 30 minutes, the mixture was sealed in a Teflon container under an argon atmosphere. It was then heated at 155 °C for 12 h to incorporate sulfur into the host materials.

### Electrochemical measurements

For the electrode preparation, the sulfur/**Fe1−xS**/**N-PCMs-900**, carbon black, and PVDF were mixed in a mass ratio of 7 : 2 : 1 in NMP solution to make a slurry. After grinding for 30 min, the slurry was coated onto carbon-coated aluminium foil *via* the doctor blade method. Then, the electrode was dried at 50 °C under vacuum for 12 h. Afterwards, the electrode was cut into 14 mm-diameter wafers. The areal loading of sulfur was around 1.0 mg cm^−2^. Coin CR2025 cells were assembled with lithium foil as the anode and Celgard as a separator in an Ar-filled glove box (UNIlab plus, M. BRAUN) with an H_2_O content of <0.5 ppm and an O_2_ content of <0.5 ppm. The 1 M LiTFSI electrolyte solution in DME/DOL (1 : 1 v/v) with 2 wt% of LiNO_3_ was used. The electrolyte volume for each cell was 40 μl. Before electrochemical testing, all the cells were aged at room temperature under an open circuit potential for 12 h to let the electrolyte wet the electrode. In the present work, the current density of 1C equals 1675 mA g^−1^. The mass of sulfur was used to calculate the specific capacity. The galvanostatic charging–discharging was conducted on a Neware battery testing system at room temperature.

### Characterization

Crystal structural characterization was conducted on an X-ray diffractometer (PANalytical X'Pert Pro) applying Cu Kα radiation (*λ* = 1.5418 Å) between 5° and 80° at a scan rate of 0.2° min^−1^. Chemical bonding characterizations were monitored by ESCALAB 250Xi X-ray photoelectron spectroscopy (XPS). The morphology of samples was investigated using a JEOL 7000F scanning electron microscope (SEM) operated at 10 kV. Samples were sputtered by a thin gold layer for 60 s before the examination. Energy-dispersive X-ray (EDX) mapping was taken on the SEM with an EDX spectrometer. The particle size was determined by transmission electron microscopy (TEM) using a JEOL JEM-2100 (JEOL GmbH, Eching, Germany) operated at 200 kV. The nitrogen sorption isotherms at 77 K were performed by the micromeritics ASAP 2020 (Accelerated Surface Area and Porosimetry system). Before the sorption experiments, all samples were degassed for 7 h at 373 K under vacuum. The specific surface area was calculated using the Brunauer–Emmett–Teller (BET) equation. Thermogravimetric analysis (TGA) was carried out at a heating rate of 10 °C min^−1^ from 50 °C to 900 °C under N_2_/air flow using a TA Instruments Discovery TG. Raman spectroscopy was performed in a Horiba Labram HR system with 532 nm laser excitation. Elemental analysis was recorded for carbon, hydrogen, sulfur, and nitrogen employing a Vario EL Elemental analyzer. ^1^H-NMR spectra were performed at room temperature using a Bruker DPX-400 spectrometer operating at 400 MHz. DMSO-d_6_ was employed as a solvent for the measurements.

## Result and discussions

The pyrolysis of porous N-rich polymeric materials is a simple and conceptually straightforward approach for the synthesis of NPCs that can replicate some specific macroscopic features of the polymer template with a certain amount of shrinkage. In the case of porous carbon membranes (PCMs), their macropores may come directly from that of the porous polymeric membranes, and the secondary micro/mesopores can be derived from the pyrolytic decomposition of the polymeric precursor.^15^ PIL has been previously utilized as the carbon precursors in the synthesis of PCMs due to their favorable physical and chemical properties, such as high thermostability and the tunable nitrogen content in the final carbon product.^4,33^ Metal components, such as cobalt nanoparticles or gold nanoparticles, have been introduced into PIL-derived PCMs previously by carbonization of a mixture of the metal salt and the porous polymer membranes.^34,35^ As a conceptual novelty in this work, the iron source **FDA** itself is a building block of the porous polymer membrane, acting as a crosslinker to ionically complex with the PIL to form the hybrid porous membrane. That is, the hybrid porous PIL membranes serve simultaneously as the template for **N-PCMs** and the source of iron sulfide nanoparticles. In this way, an extra step to introduce any metal source to the porous polymer membranes is saved.

**Fig. 1:**
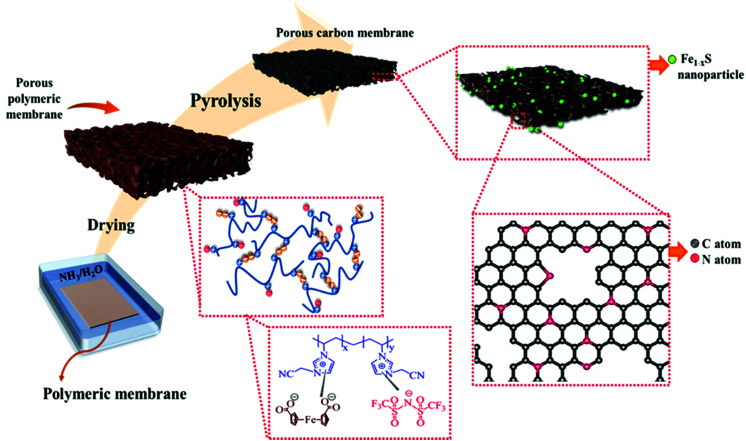
Schematic illustration of the synthetic procedure of **Fe1−xS**/**N-PCM-y** from a hybrid porous polymer membrane derived from a cationic poly (ionic liquid) and 1,1′-ferrocene dicarboxylic acid.

Fig. 1 depicts the synthetic pathway towards Fe_1−*x*_S/N-PCMs from the PIL/FDA-based hybrid porous polymer membrane. Briefly, the cationic PIL and the diacid compound, FDA were dissolved in DMSO and cast onto a glass plate to form the porous PIL membrane. A similar method was reported by us to prepare porous PIL membranes from trimesic acid.^36^ The fabricated porous polymer membrane was then converted to N-doped PCMs containing Fe_1−*x*_S (0 < *x* < 0.125)^37^ nanoparticles *via* vacuum carbonization at 900 °C. It is worth mentioning that the carbonization temperature plays a vital role in determining the physicochemical properties of the final porous carbon product. In this context, the carbonization of the hybrid porous polymeric membrane was repeated at temperatures ranging from 300 to 900 °C, so we could systematically study the effect of the carbonization temperature on the properties of the carbon products. The corresponding carbon samples were termed Fe_1−*x*_S/N-PCM-*y*, where *y* denotes the final carbonization temperature.

**Fig. 2:**
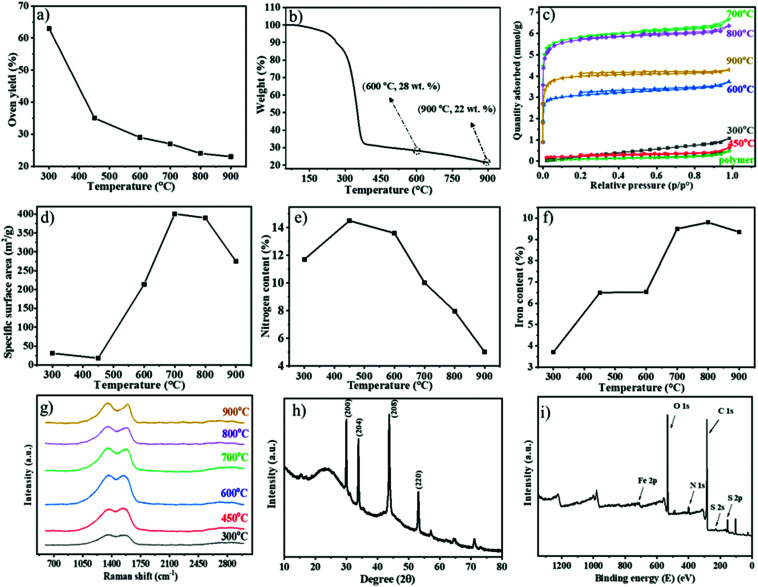
Characterization of the carbonization process of the hybrid porous PIL membrane at different carbonization temperatures. (a–g) Plots of the temperature-dependent carbonization yield (a), TGA mass residue (b), nitrogen sorption isotherm (c), specific surface area (d), the nitrogen content (e) determined by elemental analysis measurements, the iron compound content determined by TGA measurement (f), and Raman spectra (g), XRD pattern (h), and XPS survey plot of the **Fe1−xS**/**N-PCMs-900** (i).

The obtained **Fe1−xS**/**N-PCM-y** products were collected and characterized comprehensively by thermogravimetric analysis (TGA), nitrogen sorption, Raman spectroscopy, and elemental analysis. Fig. 2a shows the oven yield (the mass ratio of the residue carbon to the polymer template) as a function of the carbonization temperature. As anticipated, the oven yield decreased stepwise with rising temperatures. The most significant mass drop due to structural fragmentation occurred from 300 to 400 °C; over 400 °C, only a gradual weight loss due to structural rearrangement was seen. Through TGA, we investigated the thermal degradation of the polymeric membrane under a N_2_ atmosphere (Fig. 2b). The mass falls rapidly at a temperature between 250 °C and 400 °C, followed by a gradual weight loss above 400 °C. The massive weight loss at around 300 °C is associated with the cyclization reaction of the cyano groups of PIL, that is, any fragment that is not covalently connected to the newly formed, thermally stable *s*-triazine network will be volatilized in this stage. After that, the established *s*-triazine network will stabilize the carbon product and only lose its weight slowly with increasing temperature. This phenomenon has also been observed similarly in nitrile-containing ILs reported by Dai *et al.*^16^ In general, the weight loss according to the TGA result illustrates the same trend as the oven yield *vs.* the carbonization temperature curve in Fig. 2a. The high oven yield at 900 °C of 23% could be explained by the high thermostability of the ionic liquid-based polymeric precursor, the Fe-based nonvolatile inorganic component, and the crosslinkable cyano groups of polymers that could develop a thermally stable network during the carbonization. In parallel, an iron-free **N-PCM**, which was obtained from FDA-free porous PIL membranes, was produced as reported previously.^38^ Generally speaking, the PIL chemical structure is the key factor to control the oven yield of the hybrid porous polymer membrane.

Assisted by the TGA data (Fig. S2, ESI†), the oxidation resistance of the **Fe1−xS**/**N-PCMs-y** products towards air at different temperatures can be investigated. From 100 to 300 °C, none of the **Fe1−xS**/**N-PCM-y** samples change their weight explicitly. The rapid weight loss of samples occurred at 300 to 550 °C due to oxidative burning. For a clear comparison, the temperature at a 10% loss of their weight was plotted in Fig. S3 (ESI†) against the carbonization temperature, at which the sample was produced. The result recommended that the oxidation resistance of the **Fe1−xS**/**N-PCM-y** samples was improved at a higher carbonization temperature. That is, carbons produced at a higher temperature are more oxidation resistant.

The N_2_ sorption isotherms of the carbon samples prepared at different temperatures from 300 to 900 °C are presented in Fig. 2c. It is clear that the samples prepared at 300 °C and 450 °C are poorly or non-porous thus have little N_2_ uptake up to *p*/*p*_0_ = 1, while the samples prepared above 450 °C show a type-I isotherm, and all have significant N_2_ sorption even below *p*/*p*_0_ = 0.05, indicative of the microporous nature of these samples. The specific surface areas of the carbon products calculated by BET equation (*S*_BET_) are found to change with carbonization temperature, as plotted in Fig. 2d (data in Table S1, ESI†). The low *S*_BET_ value (<50 m^2^ g^−1^) of the products carbonized below 450 °C, illustrates the non-porous nature; however, the *S*_BET_ increases rapidly once the carbonization temperature goes beyond 450 °C. Considering the large mass loss of the precursor in the range of 250–400 °C and the formation of a *s*-triazine network at 200–300 °C, it is reasonable to judge that the polymer first builds up the thermally stable *s*-triazine network and then the fragments volatize to leave pores behind. The highest *S*_BET_ (∼401 m^2^ g^−1^) is obtained by pyrolysis at 700 °C and decreases above 700 °C, which can interpret that some of the smaller pores formed at 700 °C will start to collapse and merge into mesopores at a carbonization temperature above 700 °C.

The *S*_BET_ of iron-free **N-PCM** samples prepared at different temperatures have been reported previously.^38^

The chemical composition of the carbon products was measured by combustion elemental analysis. The nitrogen content *vs.* the oven temperature was plotted in Fig. 2e, depicting a temperature-dependent nitrogen content change in carbons. The plot disclosed that the nitrogen content first increases from 11.7 wt% at 300 °C to a maximum of 14.5 wt% at 450 °C due to the thermal decomposition of N-poor species, *e.g.* the carboxylate groups, and the continuous formation of the *s*-triazine network that is rich in N. Above 450 °C it is followed by a reduction of nitrogen content with a rapid drop after 600 °C because any nitrogen atoms, if not connected to carbon atoms in an sp^2^ hybridized state, will be mostly kicked out of the graphitic plane.^18,33,39^ The iron content of the carbon products was calculated by the mass residue as Fe_2_O_3_ at 900 °C in the TGA tests conducted in synthetic air (Fig. S2, ESI†). According to Fig. 2f and Table 1, the iron content is augmented by increments of temperature up to 700 °C, after which the iron content remains practically the same until a final content of 9.4 wt% at 900 °C. The degree of graphitization and the phase structure information of the carbons in **Fe1−xS**/**N-PCMs-y** could be probed by Raman spectroscopy (Fig. 2g). The two separate carbon peaks detected for all samples at around 1350 and 1570 cm^−1^ are assigned to D- and G-band, respectively. The disorder in carbon atoms and structural defects is related to the D-band, while the G-band can be ascribed to the ordered carbon structures.^40^ The D-band intensity was slightly higher than the G-band for all samples (Fig. S4, ESI†), indicating that the ordered and disordered structure had almost the same contribution. Similar consequences were achieved by Paraknowitsch and He *et al.* on using IL and PIL, respectively, as a precursor for producing nitrogen doped carbon materials.^33,41^ Our result could be explained by the nitrogen doping-induced local disorder, which supports the D-band intensity, as nitrogen atoms are considered as “structural defects” in the carbon matrix.

**Table 1:** Carbonization yield, specific surface area, nitrogen content determined by elemental analysis measurements, iron content, and the iron-phase structure (determined by XRD) of the carbon products prepared at temperatures from 300 to 900 °C. (am = amorphous)

Carbonization temperature/°C	300	450	600	700	800	900
Carbonization yield (%)	63	35	29	27	24	23
*S*_BET_ (m^2^ g^−1^)	31	18	214	401	390	274
N content (wt%)	11.7	14.5	13.6	10.02	7.97	5.02
Iron content (wt%)	3.7	6.5	6.6	9.5	9.8	9.4
XRD structure	am.	am.	am.	Fe_1−*x*_S	Fe_1−*x*_S	Fe_1−*x*_S

The phase structure of the iron compound was monitored by X-ray diffraction (XRD) tests of the **Fe1−xS**/**N-PCMs-y** samples (Fig. S5, ESI†). A clean Fe_1−*x*_S phase emerged once the carbonization temperature went above 700 °C. As a representative example, the XRD pattern of **Fe**_1−*x*_**S**/**N-PCMs-900** is shown in Fig. 2h, where two broad peaks located at 23.5° and 43.6° can be identified and attributed to the (002) and (100) planes of a graphitic phase, respectively. The diffraction peaks at 29.9°, 33.8°, 43.7°, and 53.1° can be assigned to the (200), (204), (208), and (220) planes of pyrrhotite Fe_1−*x*_S, respectively (JCPDS No. 22-1120).

The elemental composition and the valence states of **Fe1−xS**/**N-PCMs-900** were analysed by XPS. As presented in Fig. 2i, the typical iron 2p, sulfur 2p, nitrogen 1s, and carbon 1s peaks can be evidently detected in the survey spectrum at 715, 160, 401, and 285 eV, respectively. A high-resolution N 1s spectrum of the sample was fitted into four specific subpeaks determined at 398.38, 400.92, 399.88, and 404.48 eV (Fig. S6, ESI†), in accordance with the pyridinic (26.3 atom%), graphitic (53.5 atom%), pyrrolic (10.5 atom%), and oxidized-N (9.7 atom%), respectively.^42,43^ The C 1s spectrum certified the presence of three peaks (Fig. S7, ESI†), corresponding to the graphite-like carbon (284.65 eV), the nitrogen binding carbon (C–N, 285.2 eV), and a small peak at 287.7 eV which is the oxidized carbon (C–X).^33^ The S 2p XPS spectrum indicated three peaks at 163.8, 168.2 eV and 164.45 eV, which are in acceptable agreement with Fe_1−x_S^44^ (Fig. S8, ESI†). The high-resolution Fe 2p spectra exhibited the existence of three peaks (Fig. S9, ESI†); the two peaks located at 711.6 (39.1 atom%) and 724.9 eV (46.8 atom%) were attributed to Fe 2p_2/3_ and Fe 2p_1/2_, which can prove the presence of the Fe_1−*x*_S phase and the other peak at 716.8 (14.1 atom%) was associated with Fe^3+^.^45^

**Fig. 3:**
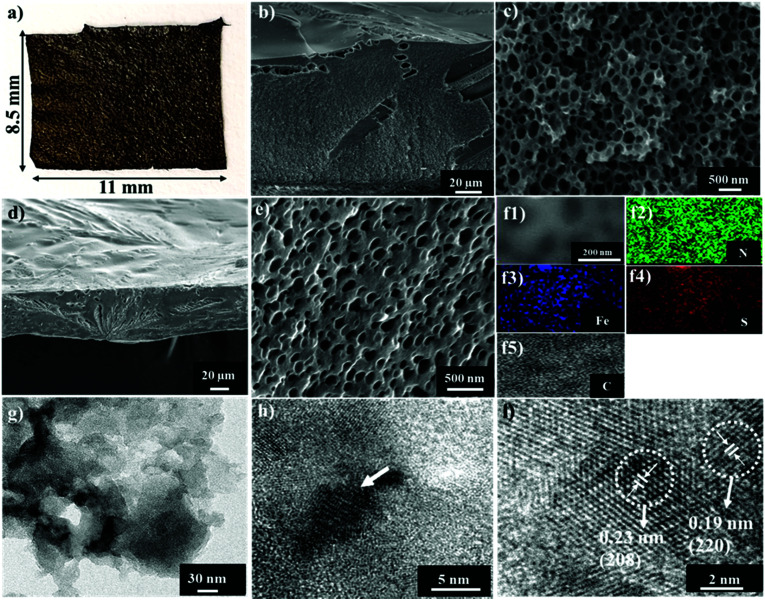
Photographs of the sample Fe_1−*x*_S-NC-900 (a), cross-sectional SEM images of the hybrid porous polymer membrane (b and c), cross-sectional SEM images of **Fe1−xS**/**N-PCMs-900** (d and e), elemental mapping of different elements in **Fe1−xS**/**N-PCMs-900** (f1–f5), TEM image of **Fe1−xS**/**N-PCMs-900** (g), and HRTEM images of **Fe1−xS**/**N-PCMs-900** (h and i).

A photograph of the final intact carbon membrane sample **Fe1−xS**/**N-PCMs-900** of 8.5 × 11.0 mm in size is presented in Fig. 3a. Since the manufacturing procedure is straightforward and simple, it can be easily scaled up to fabricate even larger ones. Fig. 3b and c represent an overview and a close view, respectively, of the cross-sectional scanning electron microscopy (SEM) images of the hybrid porous polymer membrane before pyrolysis. They revealed a three-dimensional interconnected macroporous structure with an average pore size of 262 ± 73 nm. Nitrogen sorption isotherms confirm that neither micropores nor mesopores exist in it (Fig. 2c). After carbonization, the macroscopic membrane shape is overall maintained but with a significant change in the microstructure. A high magnification cross-sectional SEM image of **Fe1−xS**/**N-PCMs-900** (Fig. 3d) confirms the membrane shape with a thickness of around 50 μm. A further high-magnification SEM image (Fig. 3e) shows the dense packing of the macropores, where the pore size distribution histogram is displayed in Fig. S10 (that of the polymer membrane is shown in Fig. S11, ESI†), showing an average pore size of 101 ± 41 nm. Keep in mind that the SEM images visualize only the macropores and large mesopores, in previous discussions around Fig. 2c, the micropores have been identified by gas sorption measurements. Combining the SEM and N_2_ sorption isotherms, **Fe1−xS**/**N-PCMs-900** combines both micro- and macropores. The bimodal pore size distribution of macropores and micropores in **Fe1−xS**/**N-PCMs-900** makes a good condition for Li–S batteries. It is a perfect environment for efficient circulation of the electrolyte's active species in the macropores, where they could readily diffuse to and from the catalytic sites packed on the micropore surface.

To extract further structural information, transmission electron microscopy (TEM) is used. In Fig. 3f, the energy-dispersive X-ray (EDX) mapping proves the microscopic uniform distribution of Fe, S, N, and C elements across **Fe1−xS**/**N-PCMs-900**. The TEM image exhibits a random distribution of the Fe_1−*x*_S, nanoparticles in the carbon membrane (Fig. 3g). A high-resolution TEM (HRTEM) image of **Fe1−xS**/**N-PCMs-900** and its enlarged view (Fig. 3h and i) show the layered texture with a spacing of 0.38 nm, corresponding to the graphitic phase. These crystalline areas are immersed in a large amorphous region, indicative of a turbostratic form of carbons with only a short-range order. At an even higher resolution, the dark dots corresponding to inorganic nanoparticles reveal the periodic lattice fringes with an interplanar distance of 0.23 nm and 0.19 nm, matching well the (208) and (220) faces of the hexagonal pyrrhotite Fe_1−*x*_S, respectively.^21^ The selected area electron diffraction (SAED) pattern measured for the Fe_1−*x*_S particles is presented in Fig. S12 (ESI†). The diffraction rings from the center toward the outside could be allocated to the (220), (208), (204), and (200) planes for the pyrrhotite Fe_1−*x*_S crystals.

**Fig. 4:**
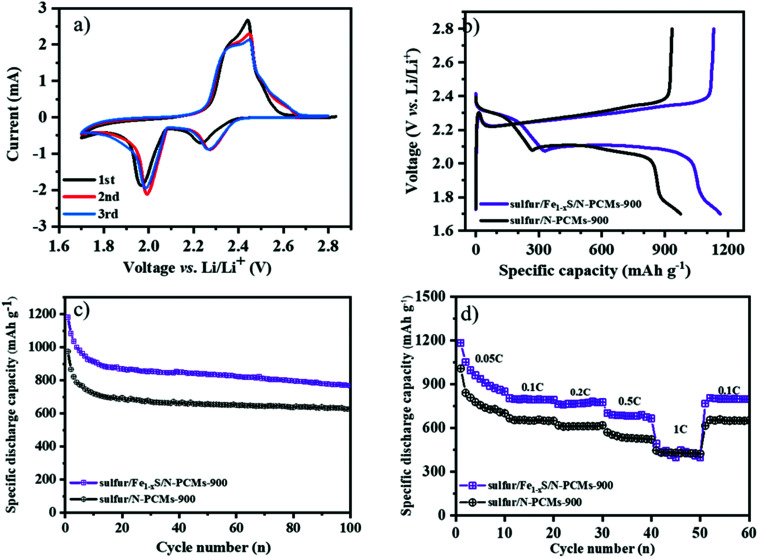
CV curves of the **Fe1−xS**/**N-PCMs-900** based cathode for Li–S batteries in a potential window from 1.7 to 2.8 V *vs.* Li/Li^+^ (a), initial discharging-charging curves (b), cycling performance at 0.1C (1C = 1675 mA g^−1^) (c), and rate capability of Li–S batteries with sulfur/**Fe1−xS**/**N-PCMs-900** and sulfur/**N-PCMs-900** as cathode materials in 1.7–2.8 V *vs.* Li/Li^+^ (d).

The multimodal porous **Fe1−xS**/**N-PCMs-900** will be a good candidate as a sulfur host material to suppress the shuttle effects of LiPSs. To test its electrochemical performance, the sulfur/**Fe1−xS**/**N-PCMs-900** composite was prepared by a melting diffusion method. The specific sulfur contents inside the composites are around 70.2 wt% and 71.5 wt% for the sulfur/**Fe1−xS**/**N-PCMs-900** and sulfur/**N-PCMs-900** composites, respectively, as seen in Fig. S13 (ESI†). Fig. 4a shows the CV curves of the Li–S batteries in the CR2025 coin cell with **Fe1−xS**/**N-PCMs-900** as the sulfur host material, which were measured in the voltage range of 1.7–2.8 V *vs.* Li/Li^+^ with a scan rate of 0.1 mV s^−1^. Two cathodic peaks can be observed at 2.23 and 1.95 V, which correspond to the reduction of sulfur to the soluble LiPSs (Li_2_S_*n*_, 4 ≤ *n* ≤ 8), and to the further reduction towards short-chain Li_2_S_2_/Li_2_S, respectively. The anodic peak at 2.50 V could be attributed to the oxidation of Li_2_S_2_/Li_2_S to sulfur.^27,46,47^ The galvanostatic charging–discharging curves of Li–S batteries with **Fe1−xS**/**N-PCMs-900** as host materials at 0.1C (1C = 1675 mA g^−1^) are illustrated in Fig. 4b. Two typical reduction plateaus are observed at 2.3 and 2.1 V in the discharge curve, which can be ascribed to the conversion reaction of S_8_ to LiPSs (Li_2_S_*n*_, 4 ≤ *n* ≤ 8) and then to short-chain Li_2_S_2_/Li_2_S, respectively. The charge curves contain only one plateau at 2.35 V, which is assigned to the oxidation of Li_2_S_2_/Li_2_S to Li_2_S_8_/S.^48,49^ The cycling stability of the sulfur/**Fe1−xS**/**N-PCMs-900** and sulfur/**N-PCMs-900** cathodes at 0.1C is shown in Fig. 4c. The sulfur/**Fe1−xS**/**N-PCMs-900** cathode delivers an initial specific discharge capacity of 1180.9 mA h g^−1^. After 100 cycles, the remaining specific capacity is 768.3 mA h g^−1^, indicating a stable cycling performance with a capacity declining rate of 0.2% per cycle. As a comparison, the sulfur/**N-PCMs-900** cathode only shows a discharge capacity of 627.9 mA h g^−1^ after 100 cycles.

Furthermore, the rate capability tests were also measured by raising the discharge/charge current density from 0.05 to 1C per every 10 cycles and returning to 0.1C. The initial specific discharge capacities of the sulfur/**Fe1−xS**/**N-PCMs-900** are determined as 1181.7, 802.4, 761.2, 701.4, and 490.5 mA h g^−1^ at 0.05, 0.1, 0.2, 0.5, and 1C, respectively (Fig. 4d), which is much higher than that of the sulfur/**N-PCMs-900** electrode at low current density. Increasing the current density leads to a decrease in the specific capacity of Li–S batteries, which could be related to the polarization caused by the poor lithium diffusion at the high current density.^50^ When the current density is changed to 0.1C, the specific capacity of sulfur/**Fe1−xS**/**N-PCMs-900** shows a durable discharge capacity of 798 mA h g^−1^, demonstrating the excellent reversibility of the electrode. The improved electrochemical performance of the Li–S batteries with **Fe1−xS**/**N-PCMs-900** as the sulfur host material is ascribed to the following features: firstly, the multimodal pore structure and well-dispersed iron sulfide (Fe_1−*x*_S) nanoparticles in the **N-PCMs** can more efficiently confine LiPSs. Secondly, the Fe**1−x**S nanoparticles can facilitate the conversion reaction of LiPSs into Li_2_S.^21^ Additionally, the existence of the macropores in the carbon material contributes to a high sulfur loading (70%) and modulates the volume changes during cycling. As a result, the porous sulfur/**Fe1−xS**/**N-PCMs-900** composite is a promising candidate as a sulfur host material for Li–S batteries.

## Conclusions

In summary, iron sulfide-contained nitrogen-doped porous carbon membranes were successfully synthesized *via* using a ferrocene-bearing hybrid porous poly (ionic liquid) membrane as a sacrificial template. The impact of the pyrolysis temperature on the final porous carbon membrane and the iron sulfide formation was studied in detail. Under optimized conditions, nitrogen-doped porous carbon composites containing Fe_1−*x*_S nanoparticles, with a nitrogen content of 5 wt% and a *S*_BET_ of 274 m^2^ g^−1^ was achieved at 900 °C. Both ordered and disordered graphitic structures were present in the nitrogen-doped carbon membrane. The nitrogen bonding states were mostly graphitic and pyridinic. The existence of Fe_1−*x*_S nanoparticles was verified by XPS, XRD, TEM, and EDX mapping methods. The nitrogen-doped porous carbon membrane loaded with Fe_1−*x*_S nanoparticles were applied as a cathode for Li–S batteries, showing the embedded Fe_1−*x*_S nanoparticles as efficient adsorbents and active sites for the conversion of LiPSs.

## Author contributions

Sadaf Saeedi Garakani and Dongjiu Xie co-conducted all experiments related to the carbonization processes and battery tests and wrote the manuscript draft. Atefeh Khorsand Kheirabad was involved in the porous polymer membrane synthesis. Yan Lu and Jiayin Yuan defined the project idea, supervised the materials synthesis and battery tests, respectively, and corrected the manuscript.

## Conflicts of interest

The authors claim no conflicts of interest.

## Supplementary Material

MA-002-D1MA00441G-s001
