# Supplementary material for: Template-synthesis of a poly(ionic liquid)-derived Fe1−xS/nitrogen-doped porous carbon membrane and its electrode application in lithium–sulfur batteries
Source: Mater Adv. 2021 Jun 25;2(15):5203–12. doi: 10.1039/d1ma00441g (PMC8328078; doi:10.1039/d1ma00441g)
Supplement: MA-002-D1MA00441G-s001 [file MA-002-D1MA00441G-s001.pdf]

Electronic Supplementary Material (ESI) for Materials Advances.
This journal is © The Royal Society of Chemistry 2021

Supplementary Information

Template-synthesis of poly(ionic liquid)-derived $Fe_{1-x}S$/nitrogen-doped porous carbon membrane and its electrode application in lithium-sulfur batteries

Sadaf Saeedi Garakani,[1] *Dongjiu Xie,*[2] *Atefeh Khorsand Kheirabad,*[1] *Yan Lu,**[2,3] *and Jiayin Yuan**[1]

[1]*Department of Materials and Environmental Chemistry, Stockholm University, Stockholm 10691, Sweden*

[2]*Department for Electrochemical Energy Storage, Helmholtz‐Zentrum Berlin für Materialien und Energie, Hahn‐Meitner Platz 1, Berlin, 14109 Germany*

[3]*Institute of Chemistry, University of Potsdam, 14476 Potsdam, Germany*

*Corresponding author:

E-mail: jiayin.yuan@mmk.su.se; yan.lu@helmholtz-berlin.de

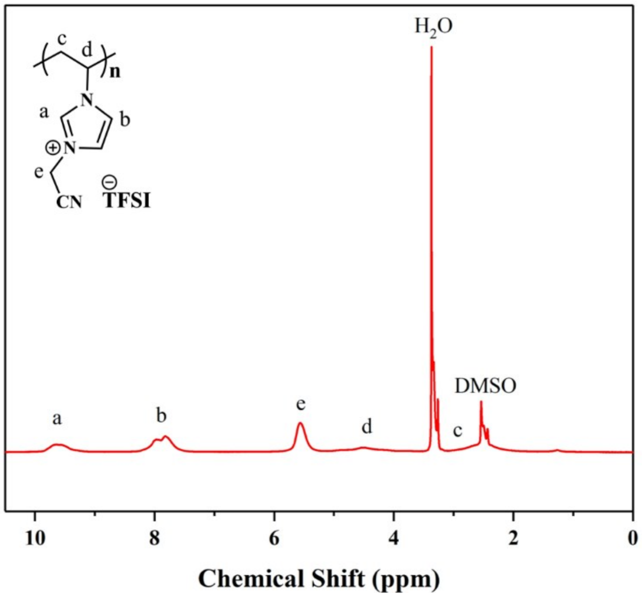

Figure S1 ^{1}H-NMR of the PIL poly[1-cyanomethyl-3-vinylimidazoluim bis(trifluoromethane sulfonyl)imide)] (PCMVImTFSI), which was used for the porous membrane fabrication.

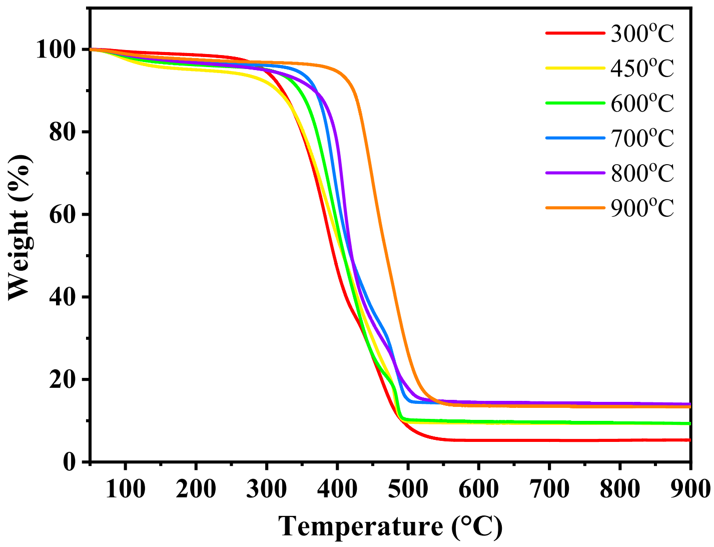

Figure S2 TGA curves under synthetic air of **$Fe_{1-x}S$/N-PCMs** prepared at different carbonization temperatures from 300 to 900 °C. The iron content of the **$Fe_{1-x}S$/N-PCMs** was analyzed by the mass residue as Fe_2O_3 at 900 °C in this test.

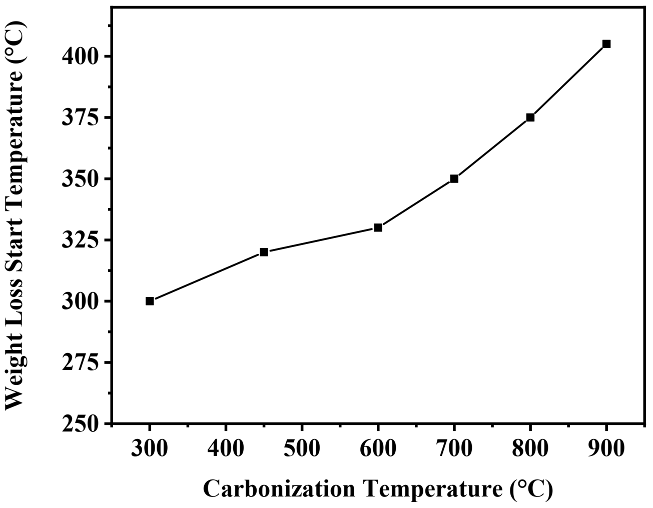

Figure S3 The temperature at the 10% loss of the **Fe_{1-x}S/N-PCMs-y** weight by TGA tests under synthetic air as a function of the carbonization temperature. The result recommended that the oxidation resistance of the samples was enhanced by increasing the carbonization temperature.

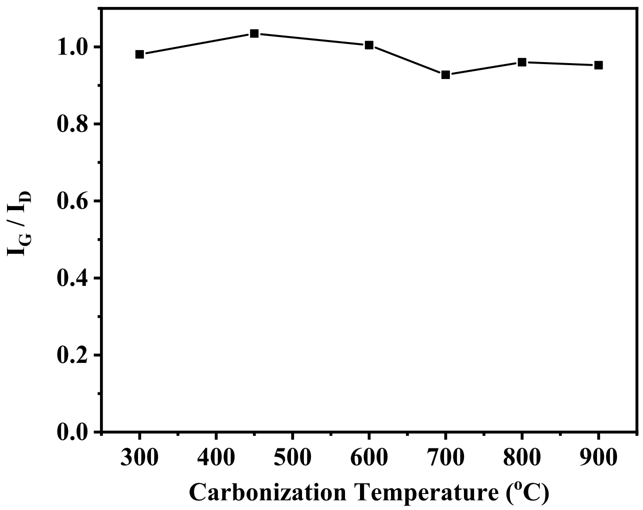

Figure S4 I_G/I_D vs. carbonization temperature of **Fe_{1-x}S/N-PCMs-y** determined by Raman spectroscopy. I_D is assigned to disorder in carbon atoms and structural defects, while the I_G can be ascribed to the ordered carbon structures.

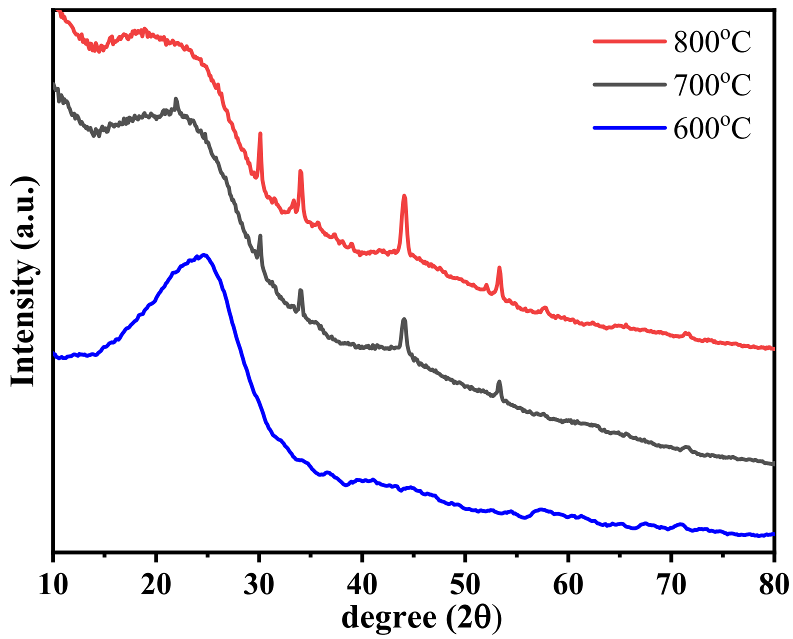

Figure S5 XRD patterns of **Fe_{1-x}S/N-PCMs-y** prepared at different temperatures.

The Fe_{1-x}S phase was not clear before the carbonization temperature went above 700 ℃.

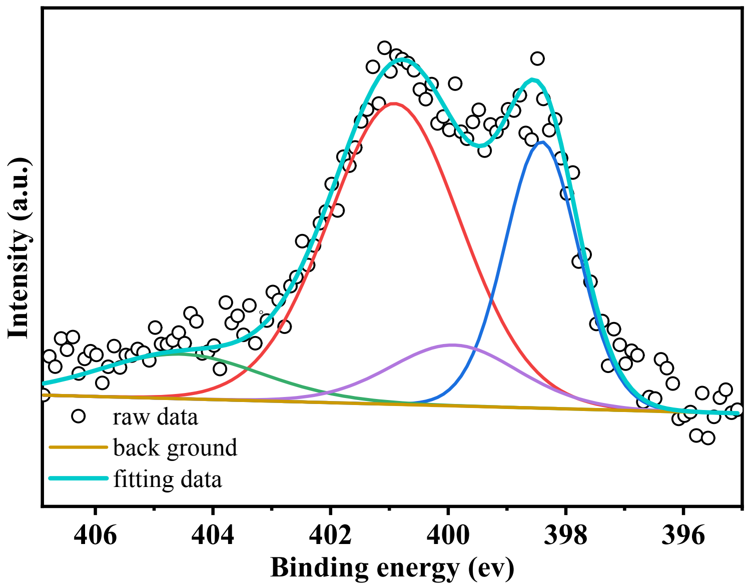

Figure S6 N1s spectrum of **Fe_{1-x}S/N-PCMs-900** determined by the XPS test.

The result demonstrates the pyridinic (26.3 atom%), graphitic (53.5 atom%), pyrrolic (10.5 atom%), and oxidized-N (9.7 atom%).

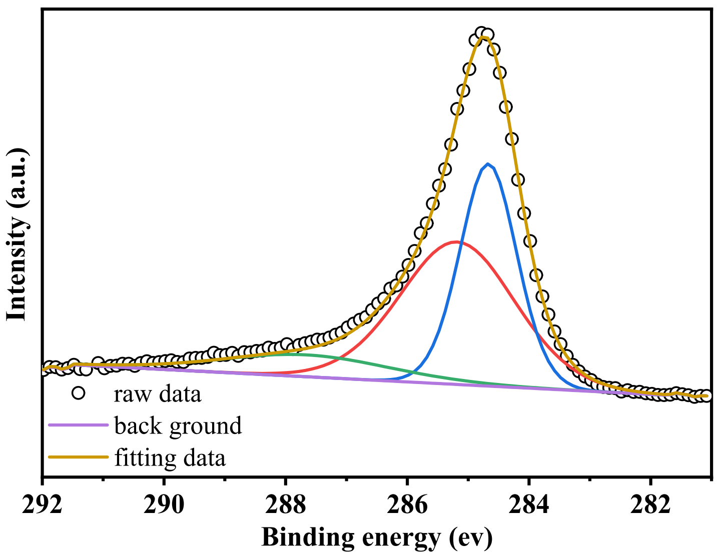

Figure S7 C1s spectrum of **Fe_{1-x}S/N-PCMs-900**.

The result proves the existence of graphite-like carbon, the nitrogen binding carbon (C–N), and the oxidized carbon (C-X).

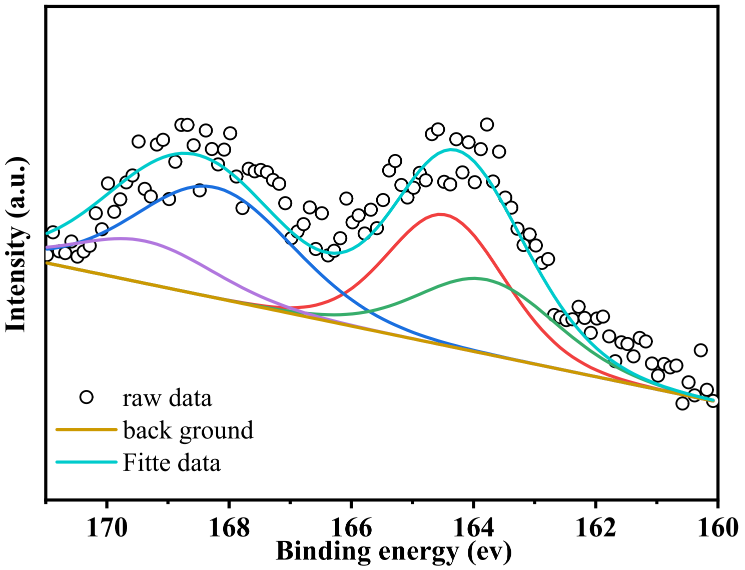

Figure S8 S2p spectrum of **Fe_{1-x}S/N-PCMs-900**. The result confirms the chemical state of S and thus the existence of the Fe_{1-x}S structure.

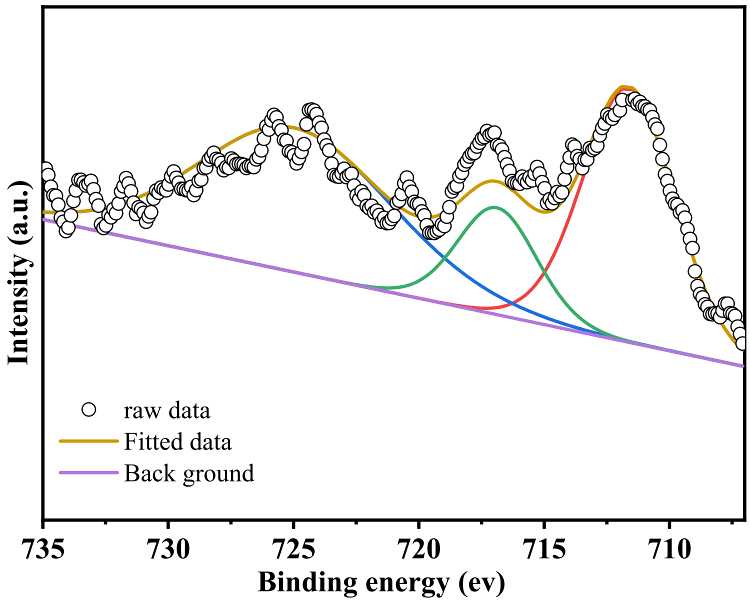

Figure S9 Fe2p spectrum of **Fe_{1-x}S/N-PCMs-900**. The result can confirm the chemical state of iron and indicate the presence of the Fe_{1-x}S phase.

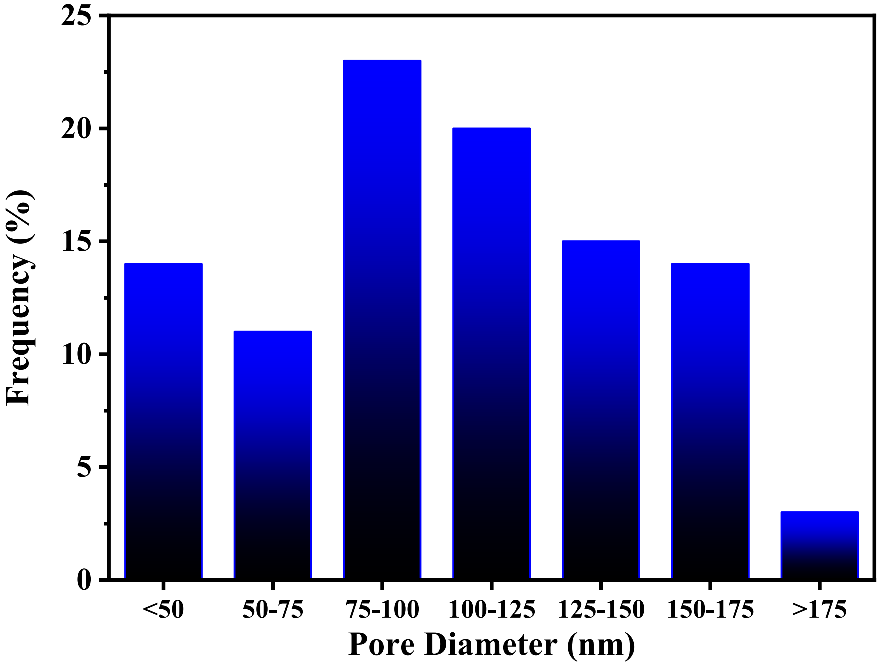

Figure S10 pore size distribution histogram of **Fe_{1-x}S/N-PCMs-900** based on cross-sectional SEM images of it.

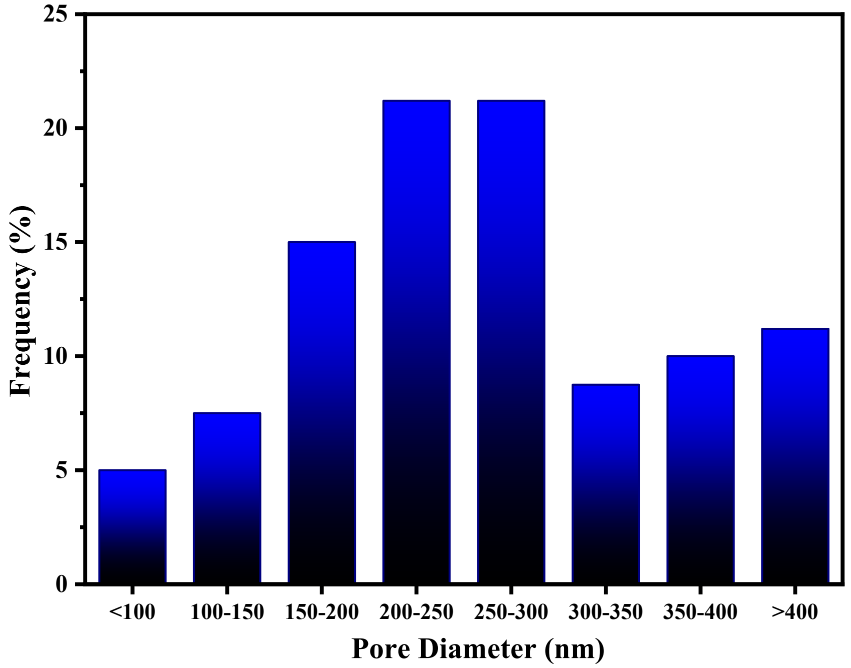

Figure S11 Pore size distribution histogram of the polymeric membrane based on its cross-sectional SEM images.

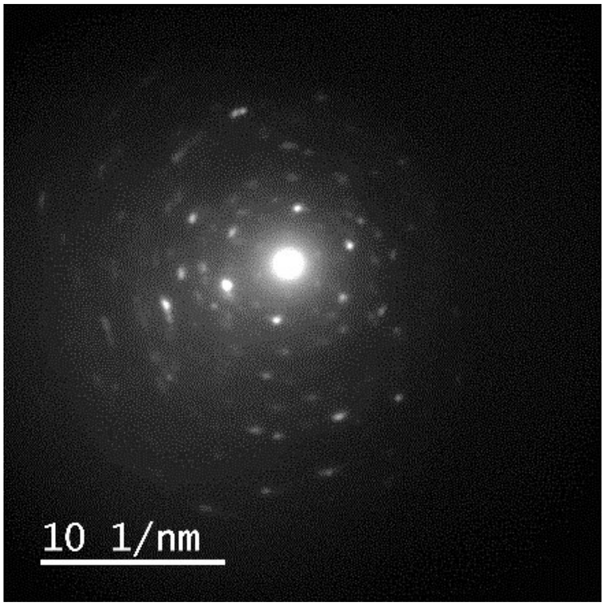

Figure S12 SAED pattern of the $Fe_{1-x}S$ nanoparticles in the **$Fe_{1-x}S$/N-PCMs-900**. The diffraction rings from the center toward the outside could be allocated to the (220), (208), (204), and (200) planes for the pyrrhotite $Fe_{1-x}S$ crystals.

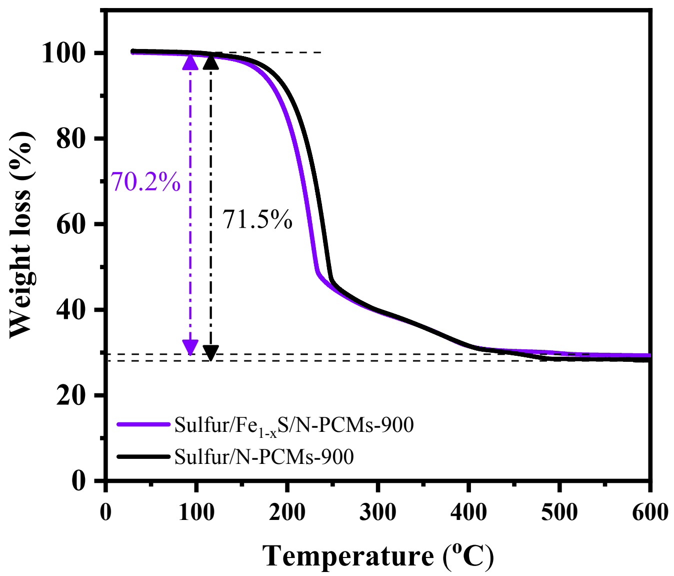

Figure S13 TGA plot of the sulfur/**Fe_{1-x}S/N-PCMs-900** and sulfur/**N-PCMs-900** composites under Ar.

Table S1 the specific surface area and porosity of **Fe_{1-x}S/N-PCMs-y** prepared at temperatures of 300 to 900 °C.

**Carbonization Temperature ℃**	$S_{BET,} m^2 g^{-1}$	$V_{total,}$ **cm³/g**	$d_{p,} nm$ ***(4V/A)***
300	31	0.036	4.65
450	18	0.024	5.3
600	214	0.20	3.7
700	401	0.37	3.51
800	390	0.38	3.3
900	274	0.26	3.8
